# Buffalo Academy of Medicine

**Published:** 1904-05

**Authors:** Wm. Irving Thornton


### Buffalo Academy of Medicine.

*Section on Medicine, February 9, 1904.*

REPORTED BY WM. IRVING THORNTON, M. D., Secretary.

The regular meeting of the medical section was held at the Academy Rooms, Public Library Building, on Tuesday evening, February 9, 1904. The meeting was called to order at 9.15

o'clock, by the chairman, Dr. A. W. Hurd. The minutes of the last meeting were read and approved.

The paper of the evening was read by Dr. Allen A. Jones, entitled,

TETANY AND ITS RELATION TO GASTRIC DISEASES.

The condition is most frequently present in children of the ages of four and five. The concensus of opinion at the present time is that tetany is due to a toxemia, probably gastrointestinal in origin. Poor hygiene, malnutrition and pregnancy are predisposing factors. It may occur as the earliest symptom of an acute infectious disease, as scarlet fever and cholera. It may be a manifestation of hysteria. In apparently infectious form it occurs occasionally in young men.

The most common pathological findings are obstruction of the pylorus, due to cicatrisation following a gastric ulcer; dilated stomach and hyperchlorhydria, or retained secretion. The hypersecretion causes hyperplasia of the gastric mucous membrane, which is followed by a weakening of the gastric walls. In one case of the writer's, there was obstruction of the pylorus, due to adhesions with the gallbladder. The treatment of these cases is surgical, pyloroplasty or gastroenterostomy being the most advisable. In the writer's opinion pyloroplasty has given better results than gastroenterostomy, provided a large enough opening is made in the former.

Dr. Edward McGuire in discussing the paper stated that by surgeons the accepted operation was gastroenterostomy, as tetany may recur after a pyloroplasty.

Dr. Colton spoke of an infant nine months old which had been fed on simply oatmeal water for several months. The child was taken with tonic spasm of the extremities. On the third day it spread upward, became general and caused the death of the infant. He believed that this case was a good illustration of the casual relation of malnutrition to tetany.

Dr. C. S. Jones reported a case of tetany in a woman 50 years of age.

Dr. Hurd stated that because of the definite symptom complex of this disease, he believed that it will prove to be of an infectious nature.

Members present, 16.

Meeting adjourned at 10.10 p. m.

### *Section on Medicine, March 8, 1904.*

REPORTED BY WM. IRVING THORNTON, M. D.

The regular meeting of the medical section was held in the Academy Room, Public Library Building, Tuesday evening, March 8, 1904. The meeting was called to order by the chairman, Dr. A. W. HURD, at 8.50 o'clock. The minutes of the last meeting were read and approved.

The paper of the evening was by Dr. HENRY R. HOPKINS, entitled,

### MALIGNANT ENDOCARDITIS OF THE RIGHT HEART.

This condition is also known by the terms, ulcerative, diphtheritic, and myotic endocarditis. The term malignant was preferred by Dr. Pepper. The right-sided form is much less frequent than the left. In 204 cases of Dr. Osler's it occurred fourteen times less frequently. The cause of this disproportion is not definitely known. The higher blood pressure of the left side is supposed to make its valves more vulnerable.

The right-sided form differs from the left in presence of pneumonic symptoms and absence of brain and kidney symptoms. The essayist reported two cases with the findings at their autopsies.

Dr. CHARLES G. STOCKTON in opening the discussion of the paper said he believed that the increased blood pressure in the left heart accounted, in part, for the more frequent occurrence of malignant endocarditis, but the fact that this condition is most frequently grafted upon a chronic valvulitis, which is usually left-sided, and the fact that certain organisms grow better in arterial than venous blood, accounts largely for the preponderance of left over right-sided forms. In concluding his remarks he reported three cases.

Dr. BLAAUW stated that in these conditions changes in the retina are to be observed, particularly hemorrhages.

Dr. BENNETT spoke of a case where antistreptococcic serum had been used, with apparent relief of the symptoms, the patient, however, dying of embolism in the brain.

The paper was also discussed by Drs. DUNHAM, LYON and HURD.

Dr. HOPKINS in closing stated that he had used collargol in two cases with no result.

Fellows present, 27.

Meeting adjourned at 10.10 p. m.

